# Canonical free-energy barrier of particle and polymer cluster formation

**DOI:** 10.1038/ncomms14546

**Published:** 2017-02-27

**Authors:** Johannes Zierenberg, Philipp Schierz, Wolfhard Janke

**Affiliations:** 1 Institut für Theoretische Physik, Universität Leipzig, Postfach 100 920, D-04009 Leipzig, Germany

## Abstract

A common approach to study nucleation rates is the estimation of free-energy barriers. This usually requires knowledge about the shape of the forming droplet, a task that becomes notoriously difficult in macromolecular setups starting with a proper definition of the cluster boundary. Here we demonstrate a shape-free determination of the free energy for temperature-driven cluster formation in particle as well as polymer systems. Combined with rigorous results on equilibrium droplet formation, this allows for a well-defined finite-size scaling analysis of the effective interfacial free energy at a fixed density. We first verify the theoretical predictions for the formation of a liquid droplet in a supersaturated particle gas by generalized-ensemble Monte Carlo simulations of a Lennard-Jones system. Going one step further, we then generalize this approach to cluster formation in a dilute polymer solution. Our results suggest an analogy with particle condensation, when the macromolecules are interpreted as extended particles.

## Abstract (web summary)

Droplet formation processes are ubiquitous in nature and accompanied by a free-energy barrier. Here, the authors present a numerical approach for a shape-free determination of free-energy barriers and demonstrate on this level an analogy between particle condensation and polymer aggregation.

The formation of equilibrium droplets from a supersaturated gas is a long-standing subject of interest, being an essential phase transition in nature^1^,^2^,^3^. More importantly, the underlying mechanism is relevant for a multitude of nucleation-like processes from statistical mechanics to material science. These include crystallization in colloidal suspensions^4^,^5^ and cluster formation in protein solutions^5^,^6^, as well as domain formation in so-called phase-change materials^7^,^8^,^9^ and glassy solids^10^. It is even connected to field theory^11^ and nuclear reactions^12^. The formal framework of free-energy calculations is straightforward, for example, in terms of reaction coordinates in phase space, but the application in computer simulations is diverse with an ongoing demand for further methodological developments^13^. A seminal application was the parameter-free estimate of crystal nucleation rates from equilibrium free-energy barriers^4^. It seems natural that the estimation of nucleation barriers becomes increasingly difficult when considering more complex systems, such as polymer or protein solutions^5^.

The rate of nucleation *R* is related by classical nucleation theory^1^,^3^ to the free-energy cost *β*Δ*F* of a nucleus on top of the nucleation barrier:


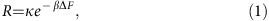


with the inverse temperature *β*=1/*k*_B_*T* and the Boltzmann constant *k*_B_. The kinetic prefactor *κ* includes the kinetic details of the nucleation process, such as diffusion and nucleus-attachment rates. The free-energy barrier may be related to the suppression in the equilibrium probability distribution. Physically relevant barriers for liquid-vapour condensation are supposed to be in the range of 20*k*_B_*T* to 100*k*_B_*T* (see, for example, ref. 14). The typical setup for the study of free-energy barriers is at fixed temperature by variation of the density or degree of supersaturation or directly in the grand canonical ensemble. The barrier is then associated with the suppression in the droplet-size probability distribution^4^ and is clearly temperature dependent^15^,^16^. This usually requires estimating the droplet size and interface, a task that introduces systematic uncertainties and strongly depends on the droplet definition. Instead, the free-energy barrier may be directly related to the volume of the critical nucleus and the pressure difference^17^, exploiting a thorough understanding of the underlying phenomenon in a clever way. In this context, the problem can be reduced to conformational phase space, knowing that canonical expectation values typically do not depend on the kinetic energy.

In the following, we address the question of how to easily obtain dependable results on nucleation barriers without invoking elaborate thermodynamic reasoning or estimating nucleus shapes. This opens the door to more complex systems with nucleation-like mechanisms such as self-assembly and aggregation, where the nucleus shapes are a priori unknown. Importantly, we consider a setup at fixed density with varying temperature—an intuitive approach from a condensed matter perspective. We focus on aggregation of polymers in a dilute setup^18^,^19^,^20^ guided by the canonical case of droplet formation in a particle gas. For the canonical case, we analyse a two-dimensional free-energy landscape and identify the energy as a suitable reaction coordinate. This allows us to formulate the problem in the microcanonical ensemble of either fixed total energy *E* or fixed potential energy *E*_p_. The first is the usual textbook definition, while the latter has been frequently applied in recent computer simulation studies. This enables us to directly discuss the effect of kinetic energy when changing between the two formulations *E*↔*E*_p_. If kinetic energy matters, only the first one allows a direct physical interpretation.

## Results

### Droplet formation free-energy barrier

We begin with the paradigm of nucleation and dissolution, the equilibrium droplet formation in a supersaturated particle gas^21^,^22^,^23^,^24^,^25^,^26^,^27^,^28^,^29^. The first-order condensation–evaporation transition separates a homogeneous gas phase from an inhomogeneous phase, where a single macroscopic droplet of size *N*_D_ is in equilibrium with the remaining vapour^21^,^22^,^23^,^24^,^25^,^26^. In fact, the probability for intermediate-sized droplets was shown to vanish^23^,^24^. In the vicinity of the transition, the energy-dominated inhomogeneous condensed phase coexists with the entropy-dominated homogeneous gas phase. A transition between both phases may only occur by energy variation upon nucleation or dissolution. In reality, this of course refers to the total energy *E*. For systems where the momentum phase space may be integrated out explicitly (see Methods section), the problem simplifies in terms of computability. We hence begin with an illustration in the potential energy formulation (denoted by a hat, for example, 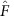) as a direct result of computer simulations before we go over to comparing both energy approaches.

**Figure 1:**
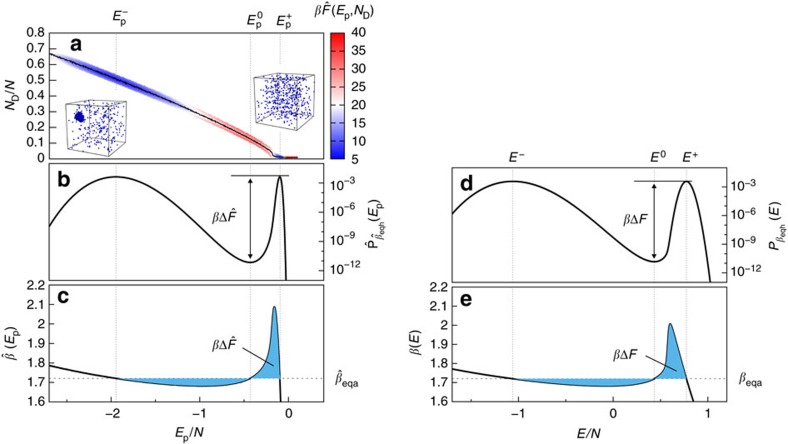
Free-energy barrier of droplet formation. (**a**) Illustration of the free-energy landscape 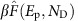 (colour map) as a function of potential energy *E*_p_ and droplet size *N*_D_ for *N*=512 Lennard-Jones particles. The minimal free-energy path (black solid line) connects a droplet (*E*_p_≈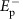) and a gaseous (*E*_p_≈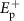) phase, visualized by the snapshots at 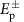. (**b**) The projection onto the reaction coordinate *E*_p_ yields the canonical potential energy probability distribution 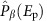, where the free-energy barrier 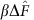 is encoded in the ratio between maximum and minimum at 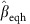. (**c**) Equivalently, 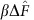 is the (equal) area size enclosed between the microcanonical inverse temperature 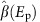 and the accordingly defined transition temperature 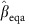, where 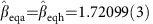. The analogous quantities are re-evaluated as a function of total energy *E* in panels (**d**,**e**) with *β*_eqa_=*β*_eqh_=1.71999(3).

Figure 1a shows the free-energy landscape 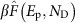 of droplet condensation–evaporation of Lennard-Jones particles (see Methods section) at the finite-size transition temperature. We define 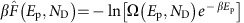, where Ω(*E*_p_, *N*_D_) is a generalization of the (conformational) density of states to the two-dimensional *E*_p_–*N*_D_ reaction coordinate space. For *E*_p_ fixed, 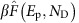 resembles a parabola with a single local minimum. The resulting transition path is shown as a black line and its relative maximum along this path (around 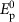) is an estimate of the transition free-energy barrier. Instead, however, one may consider the projection along the droplet size^4^ or equivalently along the energy, connected to the corresponding probability distributions 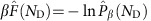 and 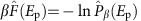.

We follow the latter approach and derive the free-energy barrier from the suppression of transition states in the canonical energy probability distributions (see Methods section) for both reaction coordinates *E* and *E*_p_. The probability distributions are shown in Fig. 1b,d and are clearly asymmetric, with a narrow peak for the gas phase and a broad peak for the droplet phase. Methodologically, both ensembles are analogous so that we limit in the following the notation to the case of total energy *E*. At the equal-height inverse temperature *β*_eqh_, 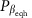
(*E*) has two peaks at *E*^±^ of equal height and in between a minimum at *E*^0^. The resulting free-energy barrier is *β*Δ*F*=ln(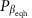
(*E*^±^)/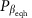
(*E*^0^)). Equivalently, one may perform the analysis entirely in the microcanonical frame^30^ and consider the enclosed area by the microcanonical inverse temperature *β*(*E*) and the canonical inverse temperature (see Methods section)


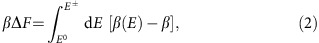


shown in Fig. 1c,e. Demanding areas of equal size yields the equal-area inverse temperature *β*_eqa_, which is in fact identical to *β*_eqh_ (ref. 31).

We notice that the energy probability distributions *P*_*β*_(*E*) and 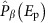 are related by a convolution involving the Maxwell–Boltzmann distribution *P*_MB_(*x*) as 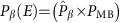
(*E*) (see Methods section). This in turn corresponds to a physical smoothing, which diminishes the ratio between maxima and minimum. As a consequence, we expect a lower barrier in the total energy formulation due to the kinetic contribution.

### Finite-size scaling of free-energy barrier

**Figure 2:**
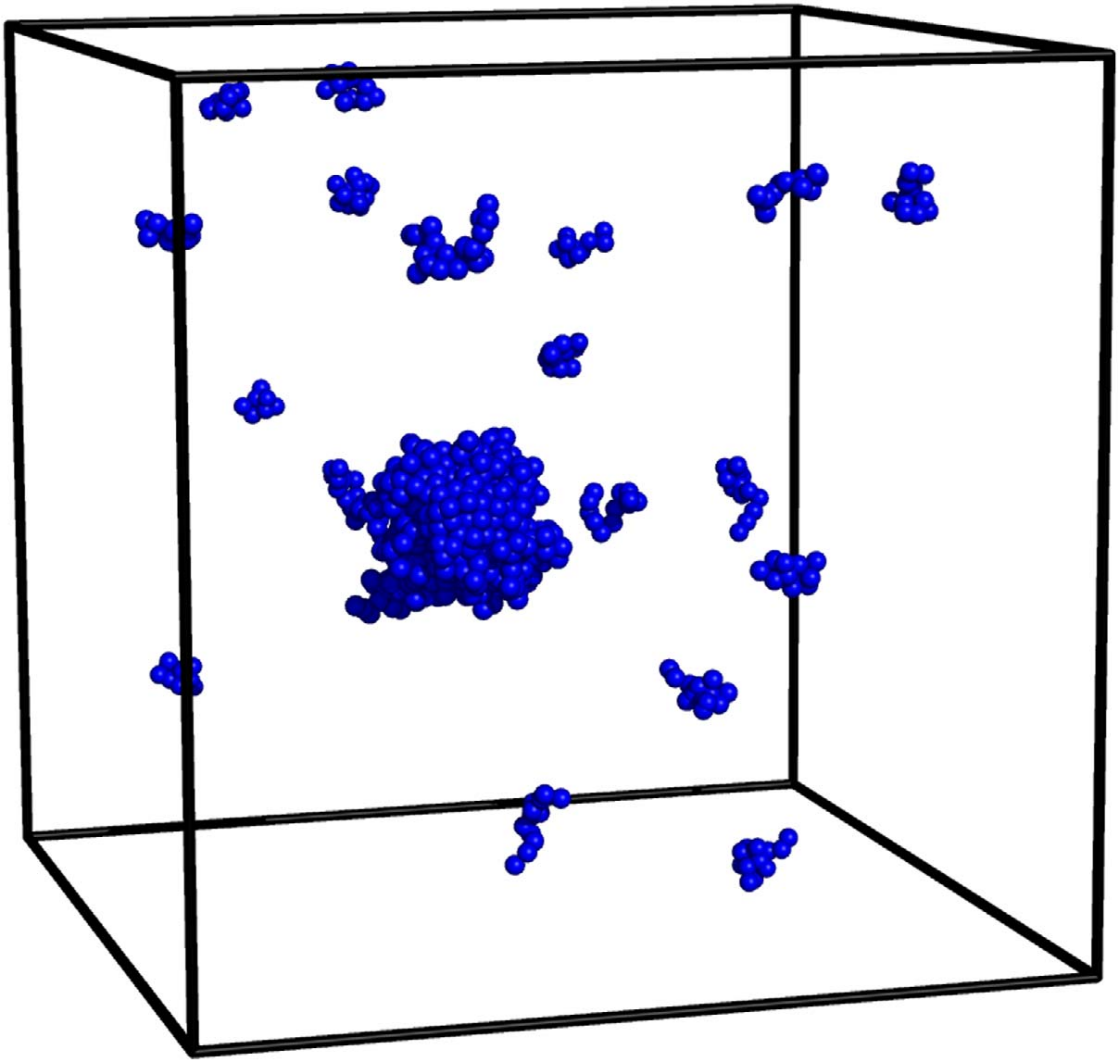
Polymer aggregate in a dilute solution. Illustration of a cluster or aggregate of polymers in a dilute solution (*N*=64 bead-spring polymers with 13 monomers each; monomer density *ρ*=10^−2^). The snapshot stems from the droplet phase (*E*_p_≈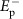).

In the following, we discuss the free-energy barrier of droplet formation in a particle gas and a dilute polymer solution as a function of system size. We here extend the notion of droplet formation to the formation of clusters or aggregates in polymeric systems. In particular, we consider linear bead-spring polymers (see Methods section), each consisting of 13 monomers. The resulting polymer cluster or aggregate is coexisting with a polymer ‘gas', see Fig. 2, a first sign for the analogy to particle droplet formation.

The free-energy barrier is commonly assumed to be proportional to the occurring interface. Here the surface of the droplet *δV*_D_ separates the liquid droplet from the surrounding gas and consequently we expect *β*Δ*F*=*σδV*_D_, with the interface tension *σ*. For any non-fractal shape, the surface area is related to the droplet volume *V*_D_ as *δV*_D_∝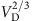. Since nucleation shows no sign of critical behaviour, this is a physically valid assumption. However, at the condensation–evaporation transition *V*_D_ itself does not scale trivially with system size *V*. At a fixed temperature, general arguments exploiting the equivalence to the Ising model imply that droplet formation is triggered by insertion of particles until a single macroscopic droplet of size *V*_D_∝*V*^3/4^ coexists with the surrounding vapour^23^,^24^,^25^,^26^. This result may be translated to a fixed-density setup using Taylor expansions, where directly at the finite-size transition temperature the analogue scaling *V*_D_∝*N*^3/4^ was verified for both lattice and off-lattice particle models^29^.

Putting everything together and introducing an effective interfacial free energy *τ*_eff_ then yields to leading order *β*Δ*F*∝*τ*_eff_*N*^1/2^. It is common for the study of interface tensions to consider logarithmic corrections^32^,^33^,^34^,^35^, dating back to early field theoretic results^11^. The physical origin are translational invariance as well as capillary waves at the interface. Altogether we use for our final scaling ansatz


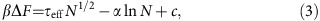


where *α* and *c* are constants. This is the leading-order exponent in equation (1). Neglecting the prefactor *κ* for now, we obtain from equation (3) to leading order the rate of equilibrium droplet formation as *R*∝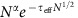. Thus for increasing system size the probability that a single macroscopic droplet forms decreases exponentially.

**Figure 3:**
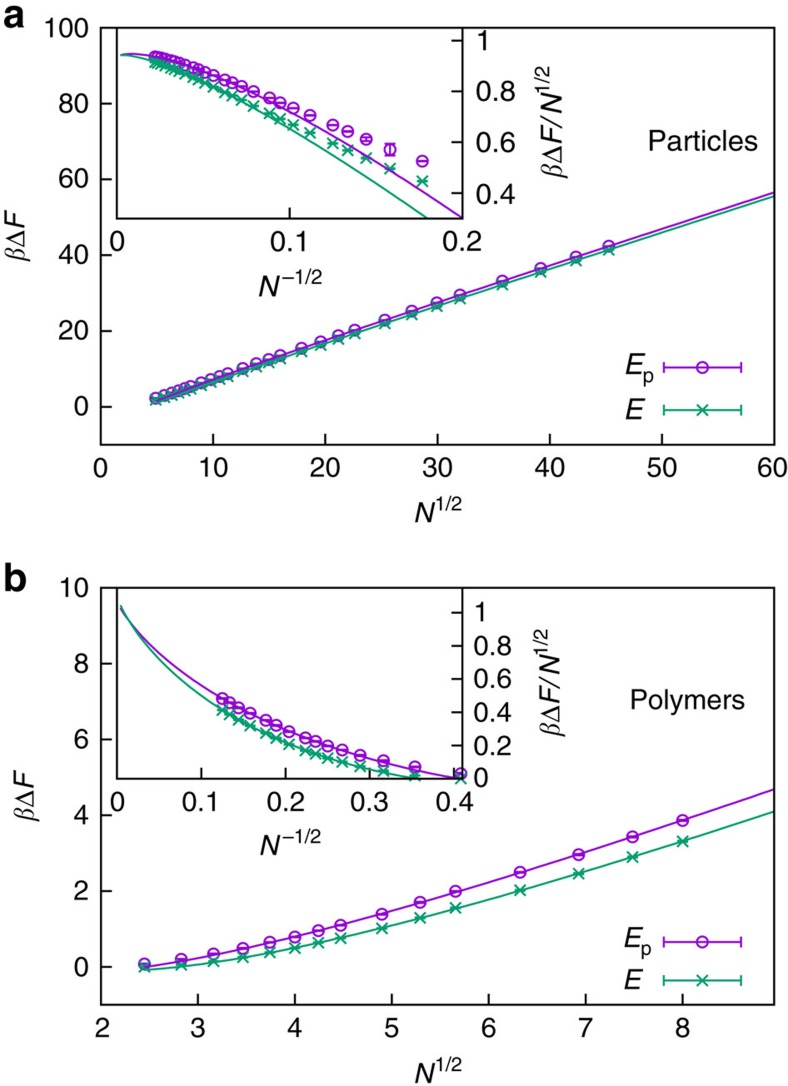
Finite-size scaling of free-energy barrier. Free-energy barriers 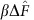 (reaction coordinate *E*_p_) and *β*Δ*F* (reaction coordinate *E*) of droplet formation in a particle gas (**a**) and dilute polymer solution (**b**) as a function of the number of constituents *N*. The leading *N*^1/2^ scaling is clearly demonstrated. The insets show the finite-size scaling of the interfacial free energy according to equation (3). Error bars indicate the s.e.m. obtained from Jackknife error analysis.

Figure 3a shows the free-energy barrier for droplet formation in a particle gas as a function of system size for both reaction coordinates *E* and *E*_p_, obtained via equation (2). Both estimates yield barriers up to about 42*k*_B_*T*, showing at close sight an almost constant shift. Only the total energy *E* includes the kinetic contribution, which is here reflected in a smaller barrier, whereas the interfacial free energies are expected to be identical, 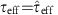. In fact, fits to equation (3) yield *τ*_eff_=0.939(4) and 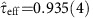, each for *N*≥*N*_min_=320 with goodness-of-fit parameter *Q*≈0.3, for reaction coordinate *E* and *E*_p_, respectively. This remains consistent within error bars under variation of *N*_min_∈[224, 1,280]. The accordance of data and fit is demonstrated in the inset. In order to test the significance of the logarithmic corrections, we considered in addition a restricted fit to *β*Δ*F*=*τ*_eff_*N*^1/2^+*c*. We obtain *τ*_eff_=0.973(2) and 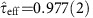 for *N*_min_=768 with *Q*≈0.2 and *Q*≈0.1, respectively. However, the estimate of *τ*_eff_ gradually decreases with increasing *N*_min_. Thus the most probable scenario remain small logarithmic corrections competing with a constant shift. The leading scaling behaviour was also observed in ref. 17 directly as a linear function of the droplet surface. The advantage of the present approach is that it avoids difficulties and uncertainties coming from the ‘correct' identification of the cluster surface.

For the cluster formation in a dilute polymer solution shown in Fig. 3b, the situation remains qualitatively similar. Fits to equation (3) yield *τ*_eff_=1.06(3) and 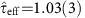, each for *N*_min_=16 with *Q*≈0.2, for reaction coordinate *E* and *E*_p_, respectively. Note that this is on the same scale as for droplet formation of particles in Fig. 3a. Compared with the particle case, the system sizes are, however, much smaller, making quantitative predictions for the polymer case less reliable. Still, the overall behaviour supports the hypothesis that cluster formation in a dilute polymer solution shows a strong analogy to droplet formation in a particle gas.

We observe that considering the kinetic contribution results in a shifted barrier. For the considered examples and relevant system sizes, the shift is about 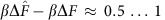, that is, of the order 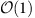. This additive contribution, while much smaller than the leading behaviour, leads to a multiplicative relation between the nucleation rates *R*∝*e*^−*β*Δ*F*^≈
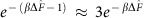
∝3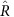. Neglecting the momentum phase space thus underestimates the rates. In common situations, however, the deviations between experiment and simulations are of the order of several magnitudes, such that the effect of the kinetic contribution may be considered subleading.

### Finite-size scaling of transition temperature

**Figure 4:**
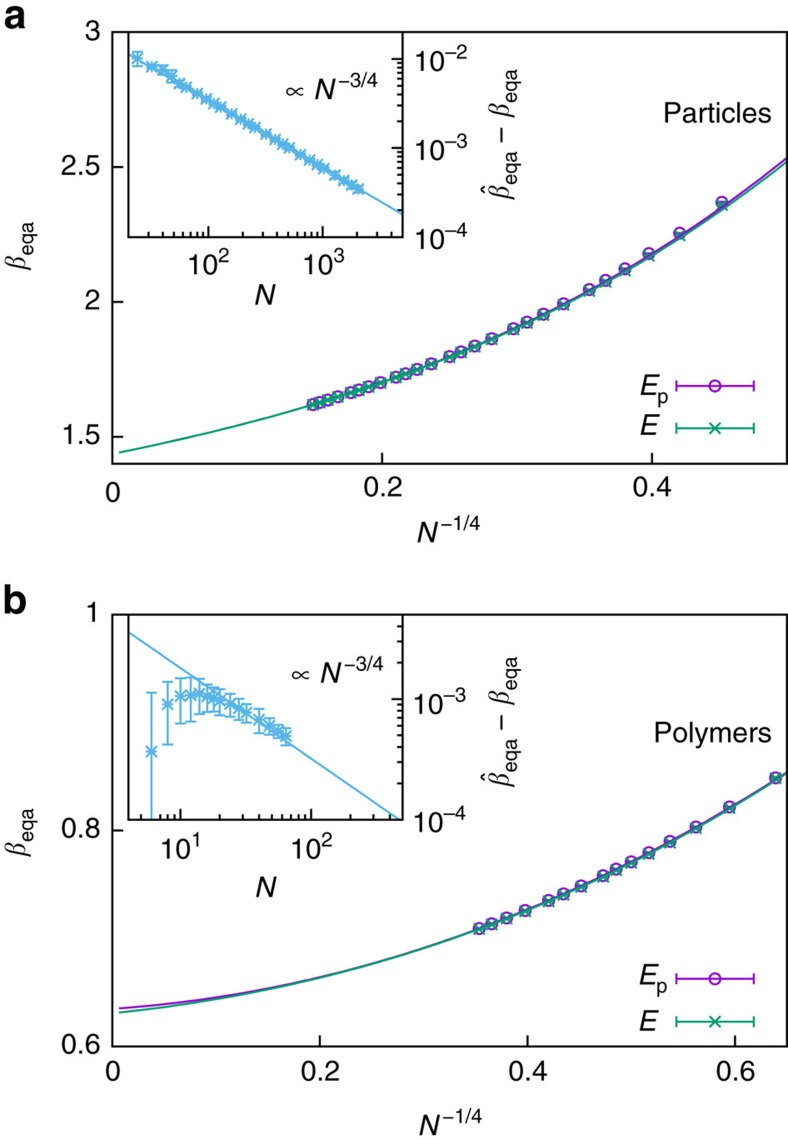
Finite-size scaling of transition temperature. Inverse transition temperature 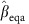 (reaction coordinate *E*_p_) and *β*_eqa_ (reaction coordinate *E*) of droplet formation in a particle gas (**a**) and dilute polymer solution (**b**) as a function of the number of constituents *N*. The finite-size scaling ansatz of equation (4) describes the data perfectly. The inset shows a vanishing finite-size difference between both ensembles 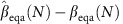
∝*N*^−3/4^. Error bars indicate the s.e.m. obtained from Jackknife error analysis.

The evaluation of the free-energy barrier via equal areas provides us with a definition of the finite-size transition temperature. At fixed density, we showed for the condensation–evaporation transition^29^ that the transition temperature, obtained from specific-heat peaks, scales as inverse power of the critical droplet radius *R*_D_∝
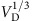
∝*N*^1/4^. The same is expected for all other transition temperature definitions. Figure 4 shows the equal-area definition together with a fit including higher-order corrections of the form


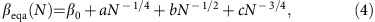


for cluster formation in both particle gas and polymer solution. In the case of particle condensation, least-square fits for *N*_min_=192 yield *β*_0_=1.436(2) and 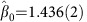 each with *Q*≈0.5, for reaction coordinate *E* and *E*_p_, respectively. The excellent fit results show that the empirical, yet physically motivated, higher-order corrections describe the finite-size scaling very well. In addition, the strong finite-size deviations open a possibility to study finite-size scaling in experiments on the nanoscale (for a conversion, see Methods section). The finite-size transition temperature of the largest system (*N*=2,048) is 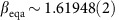, which still deviates from the thermodynamic limit by 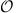
(10%).

It is worth noting that typical canonical finite-size transition temperatures, for example, the peak location of the specific heat, do not depend on the kinetic contribution to the partition function. In the canonical expectation values, the kinetic prefactor simply cancels. Here, however, we observe a finite-size difference in the transition temperature depending on whether we consider the kinetic contribution or not. Of course, the thermodynamic limit coincides. This is illustrated in the inset of Fig. 4a with a finite-size scaling of the transition temperature difference 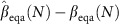. It shows a prominent power law scaling of the form 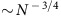, which interestingly is the same scaling as the inverse transition droplet volume. The finite-size difference arises from the convolution of an asymmetric energy probability distribution with the Maxwell–Boltzmann distribution, compare Fig. 1 and equation (8) in Methods section, which manifests in the geometric differences of the microcanonical inverse temperature and the enclosed areas in equation (2). It appears that correlations between the ensemble definitions account for compatible leading-order scaling corrections in equation (4), which further supports this ansatz and explains the observed difference.

For polymer aggregation in Fig. 4b, fits of equation (4) yield *β*_0_=0.64(2) and 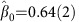 for *N*_min_=14 (guided by the inset) each with *Q*≈0.8, for reaction coordinate *E* and *E*_p_, respectively. Qualitatively, the fit ansatz describes the data already well when including the smallest system sizes. Also the finite-size ensemble deviation in the inset shows a clear *N*^−3/4^ trend for *N*≥14. Again, this is an indication for the analogy between cluster formation in polymer solutions and droplet formation in a particle gas.

## Discussion

We have presented a shape-free approach to the estimation of canonical free-energy barriers in equilibrium droplet formation. The finite-size scaling is dominated by the predicted *N*^1/2^ behaviour but we identified additional logarithmic corrections from precise numerical estimates. Somewhat surprisingly, the absolute free-energy barrier is sensitive to the consideration of the kinetic contribution. It is well known that the restriction to the conformational phase space does not influence finite-size transition points determined from canonical expectation values. These are evaluated on the level of the canonical partition function. The free-energy barrier and the associated equal-height or equal-area transition temperature, however, are determined from the energy probability distribution. Here the two formulations are related by a convolution with the Maxwell–Boltzmann distribution, which explains the finite-size differences. At the same time, the probability distributions are the integrands of the respective partition functions. In the end, this boils down to the trivial fact that equality of integrals does not imply equality of the integrands. This may become relevant once theoretical predictions and experimental measurements become precise enough. Still, a restriction to the conformational phase space retains intensive parameters in the thermodynamic limit. As a numerical advantage, considering the total energy as reaction coordinate leads to less fluctuations in the microcanonical partition function and canonical probability distribution, since the underlying convolution is a smoothing procedure of physical origin.

We provided evidence that the derived finite-size scaling of canonical droplet formation is applicable to cluster formation in dilute polymer solutions as well, despite the a priori non-trivial shape of the polymer cluster. This is a clear indication of an analogy between particle condensation and polymer aggregation. In particular, we showed that polymer clusters are in equilibrium with non-attached (free) polymers: an inhomogeneous or mixed phase of aggregate and solute polymers. This is intuitively clear when the polymers are interpreted as extended particles. The leading-order corrections then follow from the interplay between energy minimization by forming a local cluster and entropy maximization by retaining freely movable constituents. Of course, additional corrections should follow from the explicit geometry and internal behaviour of the constituents.

It is expected that the energy remains a suitable reaction coordinate for general nucleation-like mechanisms. In this case, generalized ensemble methods may unfold their full power. Moreover, our approach at fixed density provides the possibility for experiments to perform heating–cooling studies in order to probe transition rates. The presented results for polymer aggregation suggest that this approach may be generalized to studies of protein cluster formation^6^. In a wider scope, it may also find potential application for temperature-driven self-assembly^36^,^37^, crystallization in phase-change materials^7^,^8^,^9^ and glassy solids^10^, dislocation nucleation^38^ or the study of surface nanobubbles and nanodroplets^39^. Of course, experimental observations commonly include the formation of multiple clusters. Reasons for this include heterogeneities or impurities acting as nucleation seeds. We suppose that this leads to a local quasi-equilibrium on the respective length scales. Here a proper combination of the canonical droplet formation with the effect of nucleation seeds^40^ seems to be a fruitful approach to a systematic understanding. With further developments, simulations may provide reliable estimates for finite-size systems and meet experiments on the nanometer scale.

## Methods

### Microcanonical ensembles

Recently, there has been some ambiguity with the definition of a ‘microcanonical ensemble' in computer simulations^41^. This is a crucial aspect relevant for physical interpretations that appears to be unwittingly softened in the past decade. The microcanonical ensemble (*NVE*) describes an isolated system in which the number of constituents *N*, the volume *V* and the total energy *E* are conserved. Here the transfer of potential energy *E*_p_ into kinetic energy *E*_k_ and vice versa is a valid and relevant mechanism, where *E*=*E*_k_+*E*_p_. The microcanonical (Boltzmann) entropy is defined as *S*(*E*)=*k*_B_ ln Γ(*E*), with the partition function 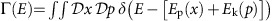, where 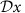 denotes the integration over state space and 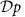 over momentum space.

The other common definition is the conformational microcanonical ensemble (*NVE*_p_), describing instead a system with fixed potential energy *E*_p_. The conformational microcanonical entropy is 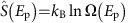, where 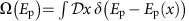 is the density of states or the conformational microcanonical partition function. The consequences are drastic: a (physical) interpretation of energy transfer from potential to kinetic energy is no longer valid. This is natural for spin systems, where a kinetic contribution is not defined in the first place (but may be exploited for numerical purposes^42^). On the contrary, it is particularly relevant for situations in soft condensed matter, for example, for particles and polymers, where interpretations of energy transfer become natural. However, there are good reasons for this choice: Ω(*E*_p_) is a fundamental property of statistical mechanics. It encodes the full information about the conformational space and allows for identification of (structural) phase transitions^30^,^31^,^43^,^44^. Furthermore, it may be exploited for reweighting techniques and flat-histogram Monte Carlo methods^18^,^45^,^46^.

The relation between Γ(*E*) and Ω(*E*_p_) is given by a convolution with the kinetic energy contribution (see, for example, ref. 47): If momenta and positions are independent, one may separate the kinetic energy contribution 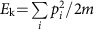, explicitly perform the momentum integration and obtain for *N* particles in three dimensions^48^


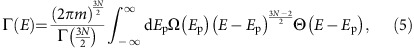


where Γ() is the Gamma function. We define Ω(*E*_p_)=0 ∀ *E*_p_<*E*_p,min_ in order to extend the integral over the full energy range (−∞, ∞). In this way, the total energy surface entropy *S*(*E*) appears as a (weighted) potential energy volume entropy. Notice that, since all Ω(*E*_p_)≥0, the classical *NVE* entropy increases with *E* and the microcanonical inverse temperature *k*_B_*β*(*E*)=*δS*(*E*)/*δE* cannot become negative, opposed to its conformational counterpart 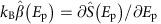. This may be an interesting aspect for a recent debate on the correct definition of entropy when connected with the phenomenological thermodynamic entropy, for example, in refs 49, 50, 51 and references therein.

Numerically, we determine the microcanonical inverse temperatures as follows. In the conformational microcanonical ensemble, we have direct access to an estimate of ln Ω(*E*_p_) (see below) such that **(*E*_p_) is obtained by a numerical five-point derivative. In the full microcanonical ensemble, we may estimate the inverse temperature in terms of microcanonical expectation values for *N* independent particles


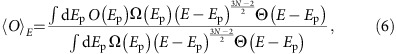


where the explicit prefactor in equation (5) cancels. Then we may express 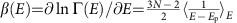.

### Canonical ensembles

The canonical ensemble is defined in terms of the partition function 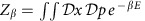, where each phase-space point is weighted with the Boltzmann factor according to the total energy. Again, the kinetic part may be explicitly integrated for generic systems. Each degree of freedom contributes with a Gaussian integral, and one obtains for *N* particles,


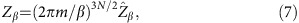


where 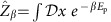 is the partition function of the conformational canonical ensemble. Both partition functions may be expressed as integrals in terms of the respective energies, namely, 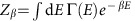 and 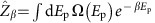. The corresponding canonical energy probability distributions are defined as *P*_*β*_(*E*)=Γ(*E*)*e*^−*βE*^/*Z*_*β*_ and 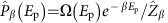.

We may now relate the two energy probability distributions by starting with the definition of *P*_*β*_(*E*) and inserting equations 5 and 7:


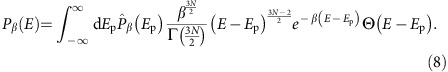


We identify the latter part of the integrand as the *N*-particle Maxwell–Boltzmann distribution *P*_MB_(*x*) and may write equation (8) as a convolution 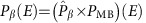.

### Lennard-Jones particles

We consider a system of Lennard-Jones particles in a dimensionless periodic box of length *L* with fixed density *ρ*=*N*/*L*^3^=10^−2^. Mutual avoidance and short-range attraction are modelled by the 12–6 Lennard-Jones potential 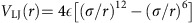 with 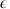
=1 and *σ*=2^−1/6^ such that *r*_min_=1. For computational efficiency, the potential is cutoff at *r*_c_=2.5*σ* and shifted by −*V*_LJ_(*r*_c_). System sizes range up to *N*=2,048, which is competitive with state-of-the-art Molecular Dynamics simulations such as well-tempered metadynamics^52^. For the chosen density *ρ*=10^−2^, a system with 2,048 particles requires a box of linear dimension *L*′≈59 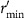
=59 × 2^1/6^ *σ*′. For argon, *σ*′≈3.4 Å such that *L*′≈22.5 nm is on the nanoscale. Of course, for a comparison to an experimental setup one should include both the explicit geometric constraints and the full Lennard-Jones potential.

### Bead-spring polymers

The considered dilute polymer solution is modelled by *N* linear bead-spring polymers, consisting of 13 monomers each, again in a dimensionless periodic box with monomer density *ρ*=13*N*/*L*^3^=10^−2^. Bonds are modelled between neighbouring monomers by the FENE potential *V*_FENE_(*r*)=−(*KR*^2^/2)ln[1−(*r*−*r*_0_)^2^/*R*^2^] with *K*=40, *R*=0.3 and *r*_0_=0.7. Non-bonded monomers interact with the same Lennard-Jones potential as above but with *σ*=*r*_0_ 2^−1/6^ such that *r*_min_=*r*_0_^18^,^19^,^20^. The total number of monomers is 13*N*, which yields 3 × 13*N* total momentum degrees of freedom in equation (5) and successive relations. The bounded bond length [*r*_0_−*R*, *r*_0_+*R*] from the FENE potential formally introduces constraints on these degrees of freedom. However, for practical applications in ordinary temperature ranges this effect is negligible and reweighting to the full microcanonical and canonical ensemble is feasible^41^.

### Multicanonical Monte Carlo simulations

Parallel multicanonical Monte Carlo simulations^53^,^54^,^55^,^56^,^57^,^58^ allow us to efficiently sample the suppressed states, by iteratively adapting an auxiliary weight function *W*(*E*_p_) to yield a flat histogram *H*(*E*_p_). The final weight function is related to the density of states up to a multiplicative factor: Ω(*E*_p_)∝*H*(*E*_p_)/*W*(*E*_p_). This gives direct access to microcanonical estimates and canonical expectation values at any temperature. Using equation (5), this even provides an estimate of Γ(*E*). Monte Carlo updates for the particle case include short- and long-range particle displacements. For updates of the polymer configurations, we employed local single-monomer shifts, bond-rotation and double-bridging moves, as well as long-range polymer displacements We measure the conformational (potential) energy *E*_p_ and the number of particles in the largest cluster *N*_D_ as in ref. 29. Error bars are obtained by the Jackknife method^59^,^60^.

### Data availability

The data that support the findings of this study are available from the corresponding author upon request. The computer code required to generate the data as well as the analysis scripts that lead to our conclusions are available from the corresponding author upon reasonable request.

## Additional information

**How to cite this article:** Zierenberg, J. *et al*. Canonical free-energy barrier of particle and polymer cluster formation. *Nat. Commun.* **8**, 14546 doi: 10.1038/ncomms14546 (2017).

**Publisher's note**: Springer Nature remains neutral with regard to jurisdictional claims in published maps and institutional affiliations.
